# Supplementary material for: FvMYB108, a MYB Gene from Fragaria vesca, Positively Regulates Cold and Salt Tolerance of Arabidopsis
Source: Int J Mol Sci. 2024 Mar 17;25(6):3405. doi: 10.3390/ijms25063405 (PMC10970457; doi:10.3390/ijms25063405)
Supplement: Supplementary file 1 [file ijms-25-03405-s001.zip › Table S1 .pdf]

Table S1 List of primers used in this study.

Primer Name	PrimerSequence (5'→3')	Purpose
AtCBF1-F	CCGACTTTGTTGGATAATATGTCCGAAG	qPCR
AtCBF1-R	TCACTAACTCCAAAGCCAGACGTCACC	qPCR
AtCOR47-F	TGGTTGTAACGGAGCATC	qPCR
AtCOR47-R	CCCCAAGAAATCAAACAA	qPCR
AtERD10-F	GCAGCAGGAGGAGAAGGG	qPCR
AtERD10-R	CACCAGGAAGAAGCCCATC	qPCR
AtDREB1A-F	ATGAACTCATTTTCTGCTTTTTCTG	qPCR
AtDREB1A-R	TTAATAACTCCATAACGATACG	qPCR
AtCCA1-F	TTTGAGGCTTTATGGTAG	qPCR
AtCCA1-R	TTTCTGAGCGTGACTTCT	qPCR
AtRD29a-F	CAACGAGGGGAAGATAAAAGTGT	qPCR
AtRD29a-R	AGCCAGATGATTTTGGAGCCT	qPCR
AtP5CS-F	GATACGGATATGGCAAAGCG	qPCR
AtP5CS-R	CCAAGTCCAAATCGGAAACC	qPCR
AtSnRK2.4-F	GAGGAAATGGGGATGCAGAT	qPCR
AtSnRK2.4-R	CGAGCCAAAGGACCATACAT	qPCR
FvActin-F	GCGACAATGGAACTGGAATGG	qPCR
FvActin-R	GACAATTTCCCGTTCAGCAGTG	qPCR
Actin-F	GTTGCCCCTGAAGAACACCC	qPCR
Actin-R	GAGATGGCTGGAAGAGGACT	qPCR
FvMYB108-F	ATGTAACGATTGGTGCCG	full-length cDNA of FvMYB108
FvMYB108-R	TCTGTGGTGGTCTTTGTCATC	full-length cDNA of FvMYB108
FvMYB108-qF	GGAGGAGCAGACGGGTTT	qPCR
FvMYB108-qR	TTGCCTAAGAGTTTGTGA	qPCR
FvMYB108-slF	GTCGACATGTAACGATTGGTGCCG	For subcellular localization
FvMYB108-slR	TCTGTGGTGGTCTTTGTCATCGGATCC	For subcellular localization
