# Supplementary figures and images for: FvMYB108, a MYB Gene from Fragaria vesca, Positively Regulates Cold and Salt Tolerance of Arabidopsis
Source: Int J Mol Sci. 2024 Mar 17;25(6):3405. doi: 10.3390/ijms25063405 (PMC10970457; doi:10.3390/ijms25063405)

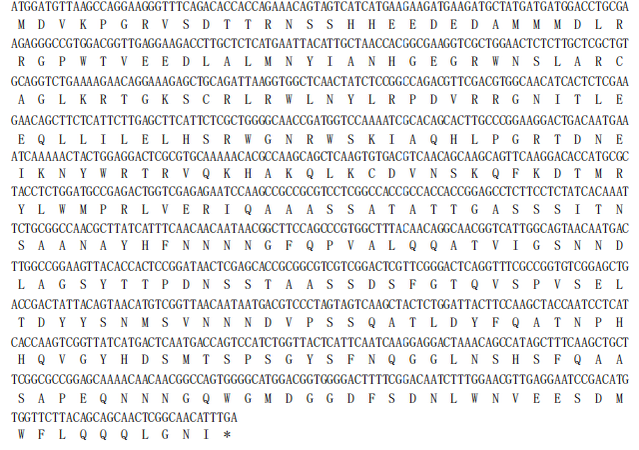

Supplement: Supplementary file 1 [file ijms-25-03405-s001.zip › Figure S1.tif]
